# The end-of-life needs of Aboriginal and immigrant communities: a challenge to conventional medical models

**DOI:** 10.3389/fpubh.2023.1161267

**Published:** 2023-07-19

**Authors:** Rosemary Leonard, Joy Paton, Peta Hinton, Sally Greenaway, Jody Thomson

**Affiliations:** ^1^Translational Heath Research Institute and School of Social Sciences, Western Sydney University, Sydney, NSW, Australia; ^2^Supportive and Palliative Care, Western Sydney Local Health District, NSW Health, Sydney, NSW, Australia

**Keywords:** end-of-life care, culturally and linguistically diverse people, Aboriginal people, palliative care, health promoting palliative care, medical system

## Abstract

**Introduction:**

Concerns have been raised internationally about the palliative care needs of migrants and First Nations people. This article presents insights from research investigating the end-of-life needs of Aboriginal and culturally and linguistically diverse people living in Western Sydney, Australia. This region has a large rapidly growing, and highly diverse population and on average low socioeconomic status. The research was guided by an advisory panel made up of representatives of supportive and palliative medicine, bereavement support, Aboriginal health, and multicultural health facilities. It aimed to generate findings to support the delivery of culturally sensitive services in the public health system.

**Method:**

The multi-method design and the conduct of the research were informed by the literature on researching with marginalized groups which highlights the ethical considerations needed to avoid replicating past injustices. Qualitative data was generated from key informants and community focus groups.

**Results:**

The analysis revealed seven themes and some suggested solutions which were relevant across several themes. The seven themes were: the Need for trusted relationships; Talking about death and dying; Knowledge of key services; Decision-making and obtaining consent from the patient; Appropriate physical spaces; Cultural practices around EOL; and Language barriers.

**Discussion:**

Within each theme a variety of cultural beliefs and practices were revealed that conflicted with mainstream medical systems, indicating the need for changes in such systems. ‘Compassionate Communities' was identified as a model to support the necessary changes.

## 1. Introduction

Access to palliative and supportive care has been recognized by the World Health Organization as a global ethical responsibility (1). Palliative care is for individuals who have a life-limiting illness and their families. It is care that focuses on improving the patient's quality of life by meeting their holistic needs (physical, emotional, psychosocial, and spiritual) in a manner that aligns with the patient's care preferences. Sometimes, this care will be known as “Supportive Care” when it is early in the disease trajectory or treats complications of the disease such as infection or metabolic issues that cause symptoms. Much of supportive and palliative care will be directed at living well in the last months or even years of life. Palliative care also involves helping the person and family prepare for the person's last weeks and days of life (the “End-of-Life” phase) and their death. It aims to ensure a death of comfort and dignity supported by high-quality care that aligns with their goals and values (2).

Concerns have been raised internationally about the palliative care needs of migrants and First Nations people. Key considerations for medical staff involve how best to communicate about the disease and prognosis, the treatment choices made, and the care goals and preferences during the EOL phase, including the death rituals and observances (3, 4). For migrant communities, there is particular concern about the marginalization of those with limited English. Some examples are Barwise et al. (5) and Abedini et al. (6) in the U.S.; Elkan et al. (7) in the U.K.; Six et al. (8) in Belgium; Nowara et al. (9) in Germany; and Sacchi et al. (10) in Italy. First Nations peoples in developed countries have reduced life expectancies, particularly from chronic diseases, and the lack of access to and take up of palliative care services is an ongoing concern [Shahid et al. (11), cross-national; Gebauer et al. (12), U.S.; Canadian Virtual Hospice (13); Gott et al. (14), N.Z.]. Australia has a dedicated *National Palliative Care Strategy (2018)* which declares that high-quality evidence-based palliative care should be accessible to all citizens. It was rated fourth out of 81 countries for its EOL care (15). However, there is a lack of detailed nationally consistent data (16), especially for urban Aboriginal communities (17), which would guide the delivery of services in local state-based health districts. The data that *do* exist show that Aboriginal and culturally and linguistically diverse (CaLD) Australians are currently under-served (18).

Theoretical analyses of modern Western health systems support concerns about the unmet palliative care needs of First Nations and culturally and linguistically diverse (CaLD) peoples. The principles of palliative care, with its holistic focus on care rather than cure for the patient and their family, stand in contrast to at least three overriding attributes of most medical systems. First, the fragmentation of care (19) contrasts with a holistic focus. Second, the dominance of mechanistic physiology (20) neglects the phenomenological experiences of the patient with its attention to cure over care (19). Third, health's implicit economic function (19) sees the institution of medicine existing to ensure citizens remain healthy enough to contribute as workers and consumers (21). Thus, people at end-of-life (EOL) especially those who are culturally or economically marginalized will not be a priority of neo-liberal health systems (21).

However, there are alternative models. Health-promoting palliative care (22) emphasizes trusting relationships and networks of care across health services and communities. This public health approach to EOL care is encapsulated in the “compassionate communities” movement (22). Here, dying, caring, and death are not constructed as solely medical events, but as social events (23–25). Moving from systems focused on mechanistic physiology and economics to compassionate communities needs, according to Rosenberg et al. (26), to be guided by three principles: re-evaluation of organizational values; recognition of the primacy of caring networks; and realignment of the inherent paternalism in healthcare provision. These are not necessarily easy changes. They are changes that need to be informed by an appreciation of, and sensitivity to, the cultural diversity of people living and dying within our communities. To that end, this article reports on research designed to amplify the cultural voices often marginalized within the context of EOL care to learn about their EOL and bereavement needs. The research was conducted in Australia and has implications for other national health systems servicing First Nations peoples and significant migrant communities.

More specifically, the research was undertaken within Western Sydney Local Health District (WSLHD). This is a large health jurisdiction with a rapidly growing and highly diverse population, many of whom live with entrenched disadvantages and significant chronic health problems. Public transport and other infrastructure have not kept pace with population growth (27, 28). The current census data (29) show that over one million people (1,1080,820) live in WSLHD. Almost 58.3% of people are non-English speakers at home. The three largest CaLD groups in the LHD are people from Mandarin (6.5%), Hindi (3.5%), and Arabic (5.3%) speaking backgrounds, which are also large language groups across the world (30). Australia's First Nations peoples comprise many culturally and linguistically diverse groups, many historically displaced from their communities in the Country, and many acculturated to urban life (31). Compared with most LHDs in Australia, Western Sydney has a relatively high number of Aboriginal people (16,614), making up 1.5% of the population. Concerningly, relatively few reach old age. Nationally, only 1.7% are over 75 years, whereas the proportion for the general population is 7.5% (32).

Researchers from Western Sydney University's Caring at End-of-Life Research Program worked with WSLHD partners to hear from the community about their culturally specific EOL and bereavement needs. The Caring at End-of-Life Research Program at Western Sydney University addresses the role of informal caring networks and their relationship with formal service providers (33), compassionate communities (34), and community death literacy (35). The WSLHD Advisory Panel consisted of representatives of Supportive and Palliative Medicine, Bereavement support, Aboriginal Health, and Multicultural Health. Together, the researchers and Advisory Panel collaborated to set clear research goals and to develop relevant research questions to achieve them. The specific aim of the research was to conduct a bereavement and palliative care needs analysis to understand (1) the end-of-life needs of Aboriginal and CaLD communities and (2) how services need to adapt to deliver culturally appropriate EOL care. The research questions were:

1. How do CALD and Aboriginal communities experience death, dying, and caring?
2. What do these communities already have that works?
3. What supports and services have they found useful, or believe could be useful, and how is this different from what is already available?
4. What other support or services do they need and who is best suited to provide this support and/or services?
5. In what ways do existing services and supports need to change to be culturally appropriate?

## 2. Materials and methods

### 2.1. Design

The research was conducted using a mixed methods design that comprised key informant interviews; culture-based community focus groups; an online Death Literacy Index survey; and in-depth personal reflection using the Photovoice method [(36, 37); See Table 1]. This multi-modal, multi-lingual, multi-disciplinary project was developed with the research partners. Aboriginal Health requested the nomenclature Aboriginal rather than Indigenous or First Nations. The research was also informed by input from 17 cultural advisors who represented the four cultural groups participating in the research (Aboriginal, Arabic, Hindi, and Mandarin). The methods followed consolidated criteria for REporting Qualitative research (38) guidelines for the conduct of qualitative research. The design and conduct of the research were also informed by the literature on researching vulnerable and marginalized groups (39–41) which highlights the ethical considerations needed to avoid replicating past injustices. Indigenous Australians and migrant communities have often been subjected to disrespectful and exploitative practices in research including a lack of significant benefit for those being researched (42, 43).

**Table 1:** Distribution of participants by gender across all methods and cultural groups.

**Participants**	**Aboriginal**	**Arabic**	**Hindi**	**Mandarin**	**Other**	**Total**
**Community focus groups**	**10**	**13**	**10**	**10**		**43**
(Gender)	(3M 7F)	(4M 9F)	(6M 4F)	(5M 5F)		(18M 25F)
**End-of-life key informants**	**2**	**5**	**7**	**4**	**27**	**45**
(Gender)	(1M 1F)	(3M 2F)	(1M 6F)	(2M 2F)	(2M 25F)	(9M 36F)
**Photo-based personal stories**	**4**	**4**	**3**	**4**		**15**
(Gender)	(4F)	(4F)	(1M 2F)	(1M 3F)		(2M 13F)
**Death Literacy Survey**	**24**	**36**	**60**	**83**		**203**
(Gender)	(3M 21F)	(13M 22F 1NA)	(27M 32F 1NA)	(21M 62F)		(64M 137F 2NA)
**Total**	**40**	**58**	**80**	**101**	**27**	**306**
(Gender)	(7M 33F)	(20M 37F)	(35M 44F)	(29M 72F)	(2M 25F)	93M 211F 2NA

From the outset, the purpose of the research was to amplify marginalized cultural voices to better understand their needs in the palliative and bereavement context. In turn, these valuable perspectives can inform policy and system change recommendations to enable culturally safe and appropriate services for people from different cultural backgrounds and those who care for them at EOL. However, in acknowledging people are the “experts” on their own experience, affording them epistemic privilege meant more than hearing their voices as expert research “subjects” (39). We sought to foster a relationship of equality and genuine participation as co-researchers and co-designers. The exchange of knowledge among the research team members and between researchers and participants ensured that the research goals would be achieved in ways that were simultaneously culturally sensitive.

We approached the research from a position of cultural humility (44) and respectful practices which focused on listening and supporting people to participate. We were inspired also by the Indigenous concept of “Dadirri” which involves reciprocity, sharing stories, “deep listening,” self-reflection, and patience [see West et al. (45)]. Normally applied in indigenist research, the principles of Dadirri are equally relevant to all marginalized people since it positions participants as equal to researchers, giving those who are normally powerless an equal voice in the research process. We ensured our collection of data was informed by Multicultural Education Officers for each cultural group and representatives of Aboriginal Health. The voice of participants was at the center of our research project, and in this article, we focus specifically on the qualitative data derived from the community focus groups and key informant interviews held with all four cultural groups. It was this data that most strongly revealed the challenges to current medical models.

### 2.2. Community focus groups

Culture/language-based focus groups with community members [see Kruger and Casey (46)] were an effective way to understand cultural differences at EOL. They also provided a space to identify possible strategies and barriers or opportunities to developing culturally sensitive EOL and bereavement services. The data were collected in 2021 and till June 2022. The onset of COVID in the initial phase of the research impacted the timing and locations of the focus groups. After the easing of community “lockdowns,” recruitment was understandably slow. People were grieving and coming to terms with the significant losses in their communities both in Australia and overseas during the pandemic. There were also ongoing concerns about health vulnerabilities which meant the integration of COVID protocols into the focus group procedures so that participants and researchers felt safe.

#### 2.2.1. Participants

Participants were adult community members from an Aboriginal, Arabic, Mandarin, or Hindi cultural/linguistic background with EOL caring experience and living in the Western Sydney Local Health District (WSLHD) catchment area. Participants were required to have cared for someone at the EOL but with a limitation of more than 6 months before the research to protect their emotional safety. English speaking was not a requirement for participants in the focus groups as interpreters were present and Multicultural Education co-facilitators were of the same language groups. The number of participants in each group ranged from 10 to 13 (see Table 1).

#### 2.2.2. Recruitment

Recruitment flyers (digital and printed) were offered in four languages (English, Mandarin, Arabic, and Hindi). Digital flyers were made available for community leaders/representatives to distribute using their email lists. Interested individuals were screened and those meeting the inclusion criteria were sent a Participant Information Sheet and provided with details of the focus group (including date, time, and location). WSLHD Aboriginal Health Unit and Multicultural Health education teams provided participants with an in-depth explanation of the project (in their preferred language) and assurance that their participation would be voluntary and anonymous. Participants in the three CaLD focus groups consisted of community members who attended the WSLHD-led health education groups. Participants in the Aboriginal focus group consisted of active members of the Aboriginal community and Aboriginal healthcare workers.

#### 2.2.3. Process

Separate 2 h focus groups were conducted face-to-face at a convenient community center with each of the four cultural/linguistic groups. The focus groups were facilitated by a researcher alongside co-facilitators from Aboriginal Health or Multicultural Health who shared the cultural background of the participants. The research assistant managed the consent forms, recording, and note-taking. After an in-depth explanation of the project aims and what participation would involve, participant information sheets in the preferred language were distributed. Following initial verbal consent, written consent was obtained and again clarified before the commencement of the focus groups, allowing regular opportunities to review consent using the Process Consent Method [see for example Dewing (47)].

The Arabic and Mandarin focus groups were both conducted in Arabic or Mandarin languages and led by a bilingual Multicultural Education Officer. A state-accredited translator was also present to help translate the discussion for the researcher. The Hindi focus group was conducted in English, as the majority of participants spoke and/or understood English. The Multicultural Education co-facilitator translated for a small group of participants who were not able to express their contributions in English. The groups began with personal introductions for all participants and facilitators including, if they wished, their EOL experience and interest in coming to the group. All focus groups were audio-recorded and transcribed verbatim. The Arabic and Mandarin focus group recordings were sent to a translator to transcribe into English and then sent to the bilingual co-facilitators to check content and accuracy. Feedback was obtained from all co-facilitators. At the end of the research analysis, all participants were invited to any of three public forums to discuss the results.

#### 2.2.4. Material

Sensitive to the fact that speaking about personal EOL experiences is a delicate matter for most of the target communities, focus groups were designed to address the subject of death indirectly. We presented a written hypothetical vignette about a person not known to the participants and they were invited to build upon that story through a series of prompting questions relating to the scenario. The vignette and prompt questions were designed to address the research aims and were prepared in collaboration with the Advisory Panel. The CaLD advisors agreed that vignettes would be an effective tool for their communities to approach the discussion. Professional translations of the vignettes were checked by the Multicultural Education co-facilitators to ensure the integrity of meaning. The Aboriginal advisors chose to use the prompt questions without vignettes to elicit discussion of participants' past experiences. Along with providing a gentle way to lead into the discussion, the vignette and prompt questions proved useful to keep the conversation topic centered on EOL and the aims of the project.

### 2.3. Key informant interviews

#### 2.3.1. Participants

Participants comprised healthcare workers within supportive, generalist, or palliative EOL care who worked within the Western Sydney Local Health District (WSLHD) catchment area; as well as other adult people working in EOL-related services. The professions of the key informants included hospital palliative care staff and managers, occupational therapists, general practice doctors and their information provider network, Aboriginal health workers, integrated community health, health educators for the multicultural community and transcultural educators for health workers, health interpreters, in-home palliative care workers, and social workers, funeral directors, death doulas, grief and bereavement counselors. Forty-nine key informants were interviewed in 43 individual interviews and 4 repeat interviews (3 interviews had more than one participant). Eighteen interviewees were from an Aboriginal, Arabic, Mandarin, or Hindi cultural/linguistic background (see Table 1).

#### 2.3.2. Recruitment

Key informants were recruited for their expertise through, or on recommendation from, the WSLHD Research Advisory Panel, Multicultural Education officers, and suggestions from other interviewees. After reviewing the initial interviews, specific informants were sought to fill the identified gaps (e.g., general practitioners).

#### 2.3.3. Process

The formally consented interviews were conducted online using Zoom. The interviewer gave a personal introduction about their role and interest in the topic. Participants were first asked to describe their roles and experiences working with the target communities. Prompt questions were used to elicit insights into the traditions and beliefs of the communities and whether death, dying, and caring were experienced differently by the majority population. Participants were invited to describe and comment on the existing services and support available and to offer suggestions for improvements, along with any recommendations for new services or supports. The interviews (of up to 2 h) were conducted in English, recorded on Zoom, and fully transcribed. The visual material was not used. At the end of the research analysis, all participants were invited to any of three public forums to discuss the results.

### 2.4. Qualitative data analysis

The de-identified key informant interviews were analyzed as one data set, and the four focus groups were analyzed separately. Each de-identified transcript was analyzed independently by at least two members of the research team. Analyzing the transcripts was an interpretative, qualitative, and data-driven inductive process that focused on emergent themes as well as specific research questions. Data were first analyzed for content according to the specific topics of the research questions. Second, the transcripts were analyzed thematically (48–50) by the research team to identify common and recurring themes, similarities, and points of difference among the cultural groups. Quotes were identified that well-illustrated the themes. Any differences between researchers were explored to better understand the data. The themes were interpreted in consultation with the WSLHD Advisory Panel to identify the thematic priorities from a cultural perspective. The Results section of this article presents the emergent themes. It was from these themes rather than from the content analysis that the challenge to conventional medical models emerged.

## 3. Results

Seven themes were identified relating to the needs of Aboriginal and CaLD people at EOL, along with some suggested solutions which were relevant across several themes. The seven themes cover needs relating to: trusted relationships; talking about death; knowledge of key services; decision-making and obtaining consent from the patient; appropriate physical spaces; cultural practices around EOL; and language barriers. Within each of these thematic areas, a variety of cultural beliefs and practices were revealed that conflicted with mainstream medical practice.

### 3.1. Theme 1: need for trusted relationships

The most consistent theme to emerge from the interviews and focus groups was the importance of relationships of respect and trust. However, there are people within the health system that insist that different cultures need to adapt to the medical culture. A key informant reported that it was not uncommon to find people who resisted adapting to different cultural needs, for example:

*If we're going to respect [them]well they have to show Health respect. You can't have it both ways … because we're in a medical institution … they need to follow the rules. And Anglos can follow the rules so why can't they?* (Volunteer coordinator)

In social contexts where there is little trust in government services and the fairness of the law, it makes sense for people to rely on trusted relationships or a chain of trusted relationships. Trust needs to be proactively developed over time:

*To develop trust, health workers need to walk with them for a while not just provide information. Health workers need to check in with their clients not just go in with a set spiel so they need to research how to approach that conversation and what they can do with the person and what they might offer or not offer that isn't going to offend them and disconnect them*. (Social worker)

Aboriginal people have experienced systematic abuse for over two centuries and still experience discrimination. They cannot ask for help and assume that they will be treated respectfully and given appropriate assistance. It is not surprising, therefore, that they want to deal with Aboriginal staff in hospitals and home care. If there are no Aboriginal staff in the relevant positions, then they would like an Aboriginal person with some authority to accompany them until the new relationship can be established:

*That first visit where I will go in and make the connection—sit down and have a chat and talk about where they're from and you know, it's quite a non-threatening visit. And then never to do an assessment at that first visit unless it's vitally important. And that's what they said, ‘If you do an assessment in that first visit, you'll lose them'*. (Aboriginal Community Nurse)

People in CaLD communities, even if they are not refugees, often come from places where there has been recent social upheaval, corruption, or few affordable services. As a result, they do not have confidence in health services here. Furthermore, although many Australians are welcoming of migrants, there is enough racism around for CaLD people to be cautious:

*in [named] Hospital, you see the doctor …she was wondering whether it is discrimination, because you can check on their face. … they just said, it's in your head, it's your mind. They couldn't find the problem*. (Mandarin focus group)

Of course, language is another concern for many. Even if they have adequate English for everyday purposes, they can be excluded by the language of the health system. So, they want to work through people from their cultural and language backgrounds and favor trusted relationships.

This focus on relationships is at odds with the health system, which focuses on efficiency and does not allocate time for relationship development. Furthermore, EOL often involves multiple visits to a hospital with a variety of symptoms over several weeks or months. People are allocated to different departments depending on the part of the body affected. Patients are unlikely to see the same doctors and nurses when they return to the hospital unless they are in a specialist unit such as Cancer Care or a Palliative Care Unit (PCU).

Although in some general practices in the community people see a different doctor each time, there are general practitioners (GPs) who have a long-term relationship with their patients. This line of communication often does not connect with the hospital as they are organized as separate systems:

*Even when there is a GP with shared culture and language, patients and families can feel abandoned when the patient goes into palliative care if the GP does not continue to follow up with them*. (Multicultural Health worker)

However, the community nurses and Palliative Care Unit staff are gradually building connections from the hospital to the GPs when the patient is at home:

*You're the medico that is looking after this patient. We're not taking over care. You're still looking after the patient and we're helping you to do that, and this is how we'll help you*. (Clinical Nurse Consultant)

Community services are also fragmented, and community workers associated with the PCU are distressed when they need to hand over their patients to an entirely different service:

*We support them through their chemo, their radiation—the whole thing. And then right at the point where they're literally dying, we have to say goodbye … And then we need to choose another service and I say, ‘Oh look (Palliative homecare service) will take you over. As of tomorrow, we're not allowed to come in' … They will get an after-hours service and doctor home visiting and that's through* (Palliative homecare service*) so there's some benefits, but the hardest thing is saying goodbye to people before they actually die*. (Aboriginal Community Nurse)

Being treated with empathy, respect, and dignity was important:

*In Syria, in our city, there's an association called “Al-Afia” they are responsible for funding major expensive surgeries such as heart surgeries. Merchants make contributions to fund this association. … They treat people with dignity… (*In Australia*) I would like for the government to take care of this in order to protect people's dignity*. (Arabic focus group)

*This paramedic was standing and telling her “Be prepared”. The mother was shocked. How can she be prepared? Is her son dying? Did he die? The paramedic said in a very rough way, without any affection, or any emotion “I didn't say this.” This is a concern regarding healthcare people. Before you treat the body, you should treat the soul. … I agree with her. This is the humane side that we miss a lot in all aspects of life in Australia. … You find that everything is so materialistic, as if they are dealing with a block and not with human beings*. (Arabic focus group)

Some respondents recognized the fundamental changes that would need to take place for health facilities to be safe respectful places for all patients:

*If you're really going to be a really respectful kind of organization, you can't just pay it lip service and have all your staff do diversity training but then the rest of the machine works exactly the same*. (Volunteer coordinator)

### 3.2. Theme 2: talking about death and dying

“*Sometimes there's this fear of talking about death and talking about grief and bereavement … and to do that well in a culturally sensitive manner is everyone's business*.” (Social Worker and Bereavement Counselor)

A range of research participants let us know that culturally, it could be challenging for people to talk about death and dying. Different cultures may have highly coded ways of undertaking these conversations, such as the term ‘Sorry Business' being widely used by Aboriginal people. For some cultures, there is a concern that talking about death could invite it in. It could feel like a betrayal of the patient because everyone was giving up on them. Or the belief that seemingly miraculous recoveries were possible meant the patient needed to hold onto hope. It caused distress when patients and carers were required to talk about their situation repeatedly with strangers in each department or service:

*When I ring an interpreter and tell them this is a palliative call, it runs in direct contrast to a lot of Muslim beliefs of ‘We don't talk about dying', we need to keep up hope for as long as possible … it does get tricky cause you're managing a culture, an ingrained cultural [belief]. (*Clinical Nurse Consultant Palliative Homecare Service*)*

*Chinese people don't do their will because they think that is a curse… But they rely on the law to do whatever after they're gone … The new generation are much, much more open*. (Health Educator Mandarin)

*My brother-in-law is a doctor. He would tell him “Your report is very good, Uncle, you don't have anything” up until the last phase when he was kept alive by the oxygen machine*. (Arabic focus group)

In contrast, one PCU manager is a strong believer in speaking clearly about death and dying:

*I don't like to fluff about because people tend to understand the word “dying.” Passing … is not something that I'm a fan of because it's not a true reflection of what is actually happening*. (PCU Manager)

A reluctance to discuss death can make it difficult to formally plan for EOL. The health care system actively promotes Advance Care Plans whereby patients identify how they would like to be treated when they are not able to make decisions. This requires a detailed discussion of bodily decline and the likely success of possible treatments. To be effective, the plans need family support lest families become distressed and seek more intrusive treatment than the patient wanted.

There are added difficulties when people rely on government income support or public housing. Such supports have specific requirements which might be attached to the dying person so there is a risk that other family members will suffer financially, or children will be taken into foster care away from their culture and community:

*it's important to have these financial conversations as hard as they are, to put in place those measures so those kids don't get taken away, the house doesn't get taken away on top of the family's grief*. (Aboriginal focus group)

### 3.3. Theme 3: knowledge of key services

Providing opportunities to develop a community understanding of palliative care was a reported need for all groups. Similarly, formal counseling and formal volunteering services are new concepts for many migrants.

#### 3.3.1. Palliative care

Some people had never heard of palliative care because it was not a service available in their country of origin. For others, palliative care was thought to be only for the last days of life, so the mention of palliative was distressing. Some Aboriginal respondents preferred the term “end-of-life” care to palliative care but those in the Hindi group all expressed that it was emotional and scary:

*end-of-life sounds more appropriate than the word palliative. I didn't know what the word meant … That's actually where the Sorry Business starts*. (Aboriginal focus group)

The idea that palliative care meant doctors were giving up was also confronting. The belief that Western medicine is infallible caused shock and anger when it failed their loved ones:

*It's a mystery how pain that started in his ears within 3 months became a problem with his kidneys. And the doctors didn't know what the issue was? Impossible!* (Arabic focus group)

One participant reported receiving no support in the home or advice from any services. She was horrified when her husband died in the Emergency Department on the floor. Other people in the group also expressed their horror at this story:

*On the floor. They didn't even give him a bed. How can a hospital do this to a human being?* … *What she said is no different to what happens in our country for the people who have no money*. … *We are here in Australia, okay? Human rights*. (Arabic focus group participants)

Some participants reported that there was an expectation that doctors should keep trying until the last breath. When this did not happen, doctors were thought to be prejudiced because the patient was not from an Anglo background. On the other hand, gratitude for the Australian health system can mean they are unwilling to ask for the things they need:

*a lot of those cultures will be—I don't know if it's grateful—like obviously the doctors looking after them so ‘thank you'. So, they're not going to ask for more because they're getting all this wonderful medical care*. (Volunteer coordinator)

As a result, patients and families were unaware of the assistance that could be given at an early stage when there is time for relationships of trust to develop and patients and families can be supported through the last months:

*a lot of people want all forms of treatment and it's when everything is exhausted, that then, you get to the point of “well refer to palliative care now” and it's almost like this person now has to accept that perhaps where they had hope, there is no hope—'I am going to die and the best I can hope is for a comfortable death'. Some people don't really accept it and some families don't really accept it, but that's the turning point and that may be really late. That's sometimes the reason we get them so late … the ones with slower progression are the ones we get referred early and these have more time to ease into this understanding of what's going on with them and they have more time to get to know our teams*. (Clinical Nurse Consultant Palliative Homecare Service)

#### 3.3.2. Counseling support

From the time it becomes clear that a person is coming to their end of life, the patient, family, and friends will start to experience grief and loss and might need support. However, some cultures do not seek out counseling support. This may be due to counseling being an unfamiliar practice or because of concerns about discussing death and dying with a stranger. Specific cultural aspects were also identified. For example, there was a belief expressed that people, particularly Hindi-speaking wives, should be suffering from their loss which made them reluctant to obtain bereavement support. The Mandarin group valued not bothering other people:

*The culture and idea for thousands of years pass on, the Chinese is privacy don't bother other people, it's my own suffering*. (Mandarin focus group)

Rules about when you need to stop grieving and move on with life meant that messages about bereavement support can be ignored after 40 days for people from Muslim traditions and 49 days for Mandarin background:

…* about the 49 days, we'd tell ourselves on that day that it's over. …it's just a token to comfort oneself. It's a legacy practice or a relic, it's meant to symbolize one's sorrow and missing of their loved one. … It's more that you spend 49 days to memorize someone, to remember someone… After the 49 days, the person departs to be on their way to Heaven*. (Mandarin focus group)

The issue of how and when to offer help with grieving at EOL is difficult in all cases. It is hard to know when people will be open to reading letters or brochures or receiving phone calls. It is aggravated by the siloing of services whereby most EOL services cease promptly at the death of the patient. Once again relationships were important and contact from someone in the health system with whom they have an existing relationship was welcomed.

#### 3.3.3. Formal volunteer services

There were numerous examples of the great work done by volunteers and ideas for the potential role of volunteers in bridging services (or siloed services). The inequity of relying on volunteers was also highlighted. Overall, volunteers were seen as a major asset especially when they come from diverse backgrounds with diverse and rare languages. Often in collectivist cultures, the idea of volunteering does not exist as there is a cultural expectation that everyone contributes to the community:

*[some] communities don't…know what a volunteer is ‘cos they don't have that word to translate. But they might help each other and be really supportive, but they don't use that [word]. So, trying to explain what we do…doesn't…translate*. (Volunteer coordinator)

Furthermore, if the concept of formal volunteers as being organized, committed, and trained is not familiar, such volunteers can be mistrusted by those from other cultures.

### 3.4. Theme 4: decision-making and obtaining consent from the patient

Obtaining informed consent from a patient for an operation or procedure is an essential ethical requirement and it is usually a routine and rapid process. Unless the patient is a child or has impaired reasoning, the medical system assumes that individual patients are best placed to make decisions about accepting treatment. However, cultural beliefs and practices add a layer of complexity to the process. In more collectivist cultures, rules about who should make decisions in the family can make it difficult to obtain consent directly from the patient:

*It's collective from the whole family (*his wife, children, and brothers*). It takes into account mainly the person's faith that they lived by, as well as their inherited norms and practices in their surrounding social environment*. (Arabic focus group)

The patient might not feel able to make that decision and there needs to be a consultation with the head of the family or more broadly with other family members. Sometimes, there can be a debate within the family about who makes the decisions:

*In the Hindi families, … I do see quite a lot of argy bargy within a family unit sometimes where say the wife might want to be the primary carer for the husband but the more educated or the more well to do person in the family might want to take over*. (Clinical Nurse Consultant Palliative Homecare Service)

Such delays can be annoying and disruptive to the hospital system which assumes patients will follow their timetable. More concerning is the possibility that the patient is not giving willing informed consent. Mandarin-speaking elders were reported as deferring to their sons who often were more fluent in English but did not confer with their parents. It was also reported that Hindi-speaking widows can believe themselves to be of such low worth that they would not be deciding based on their own best interests (Hindi community leader).

Differences were evident when focus groups were asked about the importance of maintaining life through strong medical interventions and life support systems. The Arabic group expressed such interventions were an important sign of respect and affection. The Mandarin group stated that their community had shifted toward focusing on a peaceful death:

*We say that Allah the almighty, might grant him life again. So they wait, patience, for the end of a human being's life is better. They shouldn't rush the decision*. (Arabic Focus Group)

*In the past people thought we needed to let the person hang on to their last breath, that it was filial on the part of the children to see to that. Now, our thinking is changing, people now would like the person to go peacefully, to have peace in their final leg of the journey*. (Mandarin Focus Group)

The Hindi focus group reported that a decision about turning off life support would be made jointly between the family and doctors. There is no particular cultural pressure to keep the patient alive as long as possible. A decision for the patient to die at home should be up to the patient and respected by the family, and services can come to their home to keep the patient comfortable. Several participants had not known about palliative care at home and were pleasantly surprised to find this was available.

### 3.5. Theme 5: appropriate physical spaces

The design of health facilities can be alienating. They lack appropriate symbols, food preparation spaces, and prayer rooms which would signal a welcome to diverse cultures. They often lack outside space, which was reported as a preference for Aboriginal people. Major providers of bereavement services have no strategies or staffing to assist Aboriginal or CaLD clients:

*When somebody's in the (Palliative Care) Unit and they're dying, there'll be lots and lots and lots of visitors. Like really, there could be 30 people and the expectation of the families are the fact that they will be there. They won't do anything; they won't get in anybody's way—they're really good—but the expectation by their culture and their community is that they will attend and just be there*. (Aboriginal community nurse)

Key informants reported on the needs of Aboriginal and CaLD people within the hospital setting. These needs included space for large numbers of visitors who need to come and pay their respects; a kitchen for preparing appropriate food; allowance for music, chanting, and prayers at the bedside; a prayer room with suitable furnishing and prayer mats; and appropriate art and artifacts which make people feel welcome.

Key informants who were familiar with local health facilities identified important differences between the PCU and the acute hospitals in terms of their flexibility to adapt to the cultural needs of patients and families. At the PCU, there could be larger gatherings of people, appropriate food, and appropriate rituals and artifacts. In the focus groups, the PCU was reported very positively by community members:

*We came whenever we wanted, regardless of anyone's thinking. We were there 24/7 so it didn't matter. We had people coming from the bush … And it was always overcrowded—it was like it was* ***our*** *kitchen anyway. And outside we did what we wanted to do anyway. We didn't ask*. (Aboriginal focus group)

*When the patient is still able to eat, they may wish to have something that they crave, rather than something that the hospital provides. So, this is where the family would prepare food for the patient, food that they like*. (Mandarin focus group)

### 3.6. Theme 6: cultural practices around end-of-life

#### 3.6.1. Being present at the time of death

All four cultural groups talked about the importance of visiting the dying person. In Aboriginal culture, extended family will gather, often traveling long distances. In Arabic and Hindi cultures, it is important that the dying person is never left alone while alive nor in the period immediately after death. Family and friends do not need to be specifically invited. When a traveler from India was dying, the community visited him. If they could not be beside the patient, they sat outside:

*Someone is in hospital, but family, friends, always sit outside. Always. Sister, brother, anybody*. (Hindi focus group)

For Hindi-speaking people, prayers and hymns at the bedside are important so a private room is desirable but if not, they will just draw the curtains:

*A feeling that the positive vibe goes to the patient so it's not that difficult to get out from the physical body—so their spirit comes out with no difficulties. That's our cultural perspective. All the family members recite the hymns actually and with all the prayers and the positive vibes the soul passes away*. (Hindi focus group)

The Mandarin group also reported that the patient should not be left alone and that being present at the time of death was important. It was also stated that dying at home was not desirable because of the belief that bad luck could pass on to those who used the home and visitors. However, hospitals are often not equipped to deal with a large influx of visitors:

*We all stay at the hospital to die, but at the very last minutes, the family should be around them. … Going by the current rules of the hospital, this probably cannot happen. Could the system be that if the doctor determines that the patient is now in their final moments, then could the family be quickly notified*. (Mandarin Focus Group)

There was significant additional cultural distress around this issue during our research which was conducted at the time of the COVID pandemic when hospital visits were severely limited.

#### 3.6.2. Customs around food

Food is symbolic as well as nutritious and pleasurable. Hindi background visitors always bring food:

*The well-wishers and the family members and relatives—if the patient is in a position to eat anything they definitely don't come empty handed actually. So, they come with food*. (Hindi focus group)

People from Mandarin background were known for coaxing the dying person to eat so they will not be hungry in the afterlife even when their digestive system is failing:

*Culturally food is what brings us together and so sometimes the family still want to feed the person who's dying. So that's very difficult. We struggle around that a little bit in just saying “The body is preparing to die. They don't need to eat sustenance anymore.”* (PCU Manager)

#### 3.6.3. Beliefs that care should be provided by family

Often close relatives of the patient expressed that they should be doing the caring for the patient at home especially if the patient asks to go home. This is their cultural tradition, and often there was no other option in their country of origin. However, in Australia, they might not have family and friends who can help. The situation can be aggravated by shame at accepting government services because of the belief that care should be provided by the family, even if they are not available.

At the PCU, they have the flexibility to address this by encouraging the family to bring in familiar objects and music to make the environment “more like home.” If the care of the person at home is too complex or burdensome, the PCU team may encourage the family to manage extended family/community expectations by explaining it is a medical decision to keep the patient in the hospital:

*So, you then shift your thinking, shift your care to the family member who's now feeling guilty because they (the patient) didn't get home. So, you then have to work with them, going ‘You've brought in all this. This is where* ***you*** *are. They want to be with you. You're here. You just talk to them; you play their music'. We have pictures around—they can decorate the room how they want—so it makes it as home as can be*. (Clinical Nurse Consultant PCU)

#### 3.6.4. Rituals around burial or cremation

For Aboriginal people, funerals and mourning rituals are traditionally important and extensive often taking several days or more. It is difficult for those who work for non-indigenous managers because of the lack of understanding and expectations that “Sorry business” will be dealt with minimally.

*(*Non-indigenous workers*) can go to work at 9am, go to a funeral, an Aboriginal funeral at 10am-11am and then go back to work. (*Aboriginal focus group*)*

Burial rather than cremation was important to many Arabic-speaking and Aboriginal people, but the cost can be prohibitive. There are no dedicated spaces for traditional Aboriginal burials in the city. Some focus group attendees pointed to the historical injustice of dispossession from Aboriginal land:

*why black fellas have to pay to get buried back on their own country? (*Aboriginal focus group*)*

Many Aboriginal people still have a strong connection to the part of Australia where their people (their mob) came from. Some want to return there at the end of life and others want to be buried there:

*A few people always want to go back to Country to be buried … and that's important*. (Aboriginal Community Nurse)

For Arabic-speaking people, the dead should be buried within a day, if possible, a fact that is a learning for those working in the palliative care unit:

*Islamic people last week. We knew that the person needed to be buried within the 24 h so we ensured that family knew that the person could be picked up from the unit, not go to the mortuary straight away, because we knew that those 24 h were important. So, we're learning from them*. (PCU Manager)

However, the cost of burial is a burden. It might be covered by family or friends but in Arabic culture, it is considered a loan that needs to be repaid:

*I don't want to be dependent on a person or a relative. When something happens in their family, I have to pay back the loan so in the future, it will become another financial burden on me. As a citizen, I should receive this service as part of the services provided by the council*. (Arabic focus group)

Others in the focus group agreed with this, adding that it was shameful to have to ask for money for a burial. However, there was ready community support for organizing the funeral:

*If there was a death in the family, we gather as an Arabic community. We are an emotional community, we gather, and every person will be saying I will do this, and I will do that. And they leave the family in their sorrows. They call the funeral companies, they call the mosque, and they do everything*. (Arabic focus group)

Hindi participants reported good support from the health services and their community for the funeral. Money is collected at the temple to help the family with the costs. Families reported that they can follow much of their traditional funeral practices with suitable priests in the local area and places where the ashes could be placed in the river:

*In the Hindu culture actually, it's being burned, and the ashes has been taken to the riverside—and it is our feeling that the whole soul is washed in water and so the person will be clear from the sin. That's people expectations*. (Hindi focus group)

Some migrants also wish to be buried in their country of origin and need to know that it can be organized through their consulate, but they might not know the procedure or be able to pay for the service.

### 3.7. Theme 7: language barriers

*it's not just about translating a document into a language that's comprehendible. It's translating a whole idea that might not make sense*. (Volunteer coordinator)

When people do not speak English, there is an obvious language barrier which could be assisted by having more diverse support health workers and interpreters. Interpreters tend to be available in large health facilities and are less likely to be used for counseling and community services which are away from these large facilities. Several respondents found technology very useful:

*Language is a problem, but we would use pictures and we would also ask the family to translate the words for us [there is also a] new upgrade for the Apple phone has a translation app on it automatically … [so] we can actually communicate better with them*. (PCU Manager)

Telephone interpreting services are available for health services but not for community support services. One difficulty with using telephone interpreters was that there was no opportunity to brief the interpreter beforehand about the patient's circumstances. It was reported that staff was uncomfortable about briefing the interpreter in front of the patient:

*Because some of the Asian cultures actually are very hesitant, really hesitant, about talking about death … we now drill [the interpreter] before they come in. ‘This is what you'll be talking about. You'll be talking about death and dying”. …Because we found a couple of them over the years wouldn't tell the patient. The doctor would say something and then [the interpreter] would not interpret that correctly*. (Clinical Nurse Consultant PCU)

In the context of counseling support, there was tension between those counselors wanting a direct translation of their words, and the position of interpreters concerned that ideas do not translate directly. A literal translation could have a very different meaning. Often, interpreters want to explain the significance of what is being said, for example, why the family feels they need to feed the patient. In some instances, there was a lack of trust in interpreters based on this issue:

*Another dilemma for interpreters is if they are asked if they know of any relevant services, when their role is to simply interpret the words except for the specific cultural-related information. They are not allowed to give personal opinions, but it is difficult to always tell what is culture-specific and what is personal opinion*. (Medical Interpreting Service Manager)

Using interpreting services might also make it difficult to connect with someone more deeply on the level that is needed for counseling. As a result, working with interpreting services on very sensitive matters can be seen as a barrier. The Mandarin focus group participants all had a phone number that gave them direct access to interpreters. However, being one of the most populous migrant groups and knowing there are many doctors and nurses of Chinese background, they expressed a preference for hospitals to put some effort into allotting them, Chinese staff.

With oral and written communications, for example, developing a factsheet, it is not enough to translate from English into another language. Culturally appropriate inclusive language is needed, also recognizing that sometimes this language cannot translate in any straightforward sense. Some roles and activities do not have a translation for other cultures. As already noted, some migrants are not familiar with the concepts of palliative care, volunteering, or counseling so these terms need the skill to translate, and more extensive explanations might be needed.

Language differences can be used as a guide to augment current services. For example, where it is necessary to slow down and explain what is happening in the current palliative situation. It is a reminder that medical language and the jargon in the health services are often not clear to those of non-English-speaking backgrounds:

*Even if you're born here, you're raised in Australia, the health system in itself is overwhelming let alone with a terminal illness*. (Clinical Nurse Consultant PCU)

*The doctors are very good, and they try really hard but once you give over a couple of pieces of information [to the patient or family], the rest just goes in one ear and out the other. Just bounces off. They can't absorb it all. So, they go home and think about and they may ring back, and we discuss it. It is very overwhelming and culturally for a lot of people … it's a shock … with anybody, it's not just people who don't have English as their first language*. (Clinical Nurse Consultant PCU)

### 3.8. Suggestions for addressing the problems

The antidote for many of the sources of distress identified in the above themes was communication and the development of trusted relationships where people are treated as whole persons, not as health conditions. A successful model trialed during the period of this project was employing an Aboriginal Supportive and Palliative Care Worker who comes from the local community. They guide patients and families both within the hospital and in the community, standing up to the health establishment when necessary. Respondents who knew about this role were highly appreciative and those working with CaLD people wanted similar specialist workers:

*We just need more Nicoles!* (Culturally specific supportive and palliative care worker)

Respondents from interviews and focus groups suggested that community education about EOL and palliative care was needed. To date, the focus of Multicultural Health education teams has been topics requested by communities, and no one had asked for information on palliative care. Indeed, they doubted that anyone would attend an information session due to the cultural resistance to talking about death and dying. However, younger generations are more open, and they could learn more about how to care for their parents. Furthermore, caring in the family is valued so information about palliative care framed as caring could be appreciated.

More diverse and extensive volunteer programs were also suggested. The valuable role of volunteers was recognized especially their freedom to work across health silos and into the community. Indeed, health staff were regretting the lack of volunteers under COVID restrictions. Suggestions included reaching out to communities to recruit volunteers from a wide variety of cultural backgrounds to support the diversity of patients. This includes people willing to be on call to help patients with specific language needs and creating a register for hospital staff. Therefore, a volunteer coordinator is present at each hospital to provide training and support, particularly in CaLD perspectives. Cultural training on Aboriginal perspectives is already a requirement. Furthermore, volunteers need training to understand the roles of social workers, community nurses, and others working in the EOL space so they could better coordinate their efforts. In return, the volunteers should be recognized as an integral part of the hospital team:

*They do the 4 days of training when they start and then they continually do training … they become more Health Literate, so then when they go back to their own families or communities that they can share that insight... I see volunteer service, well particularly my volunteer service, as quite specialized and educated and literate*. (Volunteer Coordinator)

In addition to the hospital-based volunteers, numerous community and religious groups can provide voluntary support. Staff would like an up-to-date and thorough register of Aboriginal and CaLD community support people and services such as not-for-profit groups and religious leaders.

On the issue of funeral expenses, funeral funds were found to be expensive scams whereby people lost everything if they missed one payment. One respondent said his church had a donation box for the costs at every funeral. Another practical suggestion was an agency that gave loans for that purpose:

*Regarding the funeral services, I say if any association or agency can financially help the family of the deceased. … because death happens suddenly. So, if we can pay them through installments that would be great*. (Arabic focus group)

If these seven themes and suggestions were taken on board by the health and community services, then mainstream medical services would need to reexamine their orientation and priorities and be prepared to change.

## 4. Discussion

This research offers important insights for the service providers delivering care for people in the last months, weeks, and days of life; similarly for people and groups advocating for the provision of more culturally appropriate palliative and EOL care for their communities. Here, we acknowledge some of the limitations of the study before discussing the evident interrelationships among the research themes identified. The connections between them reinforce the challenges in delivering culturally appropriate services. We also consider some of the practical suggestions for improving EOL care. These include ways to develop trust with people; spatial considerations that also extend the flexibility of care evidently in some palliative care units to other locations and areas of the health service; having dedicated workers with requisite cultural and/or language skills; and ways to develop community knowledge about EOL services. Finally, we return to Rosenberg et al. (26) three principles for Compassionate Communities. We consider what health services might need to do in re-evaluating their organizational values; acknowledging the primacy of caring networks; and transforming the paternalism of health systems.

### 4.1. Interrelationships among the thematic findings

Notably, yet perhaps not unexpectedly, there is an identifiable interrelationship among the themes in the results. The first three themes tell us that it is important for people to know and trust care providers; there are cultural limitations around discussing death and dying; and there are relatively low levels of knowledge about palliative care and the various services available to assist with care in the last months, weeks, and days of life. The interplay between these three themes is shown to contribute to reduced access to culturally appropriate quality palliative and EOL care. There is a negative cycle among the three themes whereby not talking about death and dying leads to limited knowledge about palliative care. In turn, this meant that patients and families lost the opportunity to develop the relationships of trust which were so important to them. Language barriers cut across the seven themes, aggravating all other issues.

### 4.2. Suggestions for ameliorating the problems

The research participants had a wide range of practical suggestions for improving EOL care for people from Aboriginal and diverse cultural groups. Many of these suggestions would be valuable for all people in need of EOL services. However, it is important to recognize that there are additional barriers for Aboriginal and CaLD people. Barriers to trust can be one of the most entrenched obstacles in the delivery of all forms of healthcare including palliative and EOL care. The treatment of First Nations peoples and migrants, especially refugees, by many governments, including Australia, is not a record that engenders trust. Migrants might also have had negative experiences with the government in their country of origin. Trust requires a strong relationship with people who can guide them and help them understand the strengths and limitations of the services on offer. Often, this needs to be someone from the same cultural background. Sometimes adult children have the education and resources to fulfill this role or a close relationship with their family doctor is another path for long-term migrants.

Spatial considerations can also be important in building trust. Appropriate spaces with visual symbols and messages in multiple languages help to develop trust by sending the message that Aboriginal and CaLD people are welcome and valued. So too, when spaces allow for the expression of culture. The aspects here are wide-ranging. They include the need for adequately sized spaces that can accommodate family and visitors of sometimes very large numbers; sensitively located spaces that can accommodate cultural rituals, music, or chanting; and dedicated areas for food preparation and consumption. In this research, the only place in the healthcare system identified as possessing flexibility of care allowing for family visits, with suitable food, music, significant images, and rituals, was the palliative care unit. Yet, this flexibility is still very much needed in other parts of the health and community service systems. Given the diversity of places where people die—emergency departments, hospital wards, nursing homes, ambulances, and their own homes—the clear implication is that many more services need to consider a more flexible, culturally safe approach to care at the EOL. An effective palliative care model can pave the way for how the existing health services might respond to culturally specific needs at EOL.

The deployment of dedicated workers, not only with diverse languages but also from the cultural backgrounds of those accessing palliative care and bereavement support, is an urgent need. In the research, there was a clear expression of support for such roles. The new Aboriginal Supportive and Palliative Care Worker demonstrated that many of the problems of understanding the health system, bridging silos in services, and developing trust can be effectively addressed through their role. No doubt that culture-specific Supportive and Palliative Care Workers could help CaLD communities too. However, there are inevitable questions about the feasibility of this suggestion. The provision of culture-specific workers for all of the 170 different language groups in Western Sydney would be costly. Is it viable to provide one Multicultural Supportive and Palliative Care Worker who would coordinate the needs of all CaLD people in the LHD? Could there be several to cover the large language groups? Could people from diverse backgrounds already working within the health system be trained as “care navigators” who can guide others around the system. Given that CaLD people make up over 50% of the WSLHD population, there is a strong case for many more culturally specific workers, and this would assist with improving the equity of palliative and EOL care to significant sections of a diverse community.

Another challenge is presented when considering how to provide culturally appropriate information and education about palliative and EOL care. Many people from culturally diverse groups do not want to talk about EOL, at least not outside their cultural norms. This may also be due to wanting to “hold on to hope,” wanting to avoid summoning death by considering it or talking about it, or just being uncomfortable with the topic, as is the case for many in the general population, too. The literature is clear that we cannot engage in cultural stereotyping when considering how best to address EOL and palliative care needs in the setting of life-limiting illness (51). Skillful communication in the setting of a trusted therapeutic alliance is especially important in these cases. Furthermore, considering local strategies within cultural groups to provide information and answer questions about service provision in the setting of age and illness may be a good starting point for conversations about how to best provide EOL information in a culturally safe way.

Mainstream Western culture has encouraged planning and open discussion around dying and provided information about advance care planning, services available, and how to ensure a death that minimizes distress for patients and families. Tapping into existing community programs such as Dying to Know Day and “Death Café's” makes the conversations more normal; less taboo and shocking. Culturally acceptable forums within local community events might serve the same purpose. A strategy to make the education suggestions feasible is to tap into existing resources including, multicultural health services, palliative care education providers, and extending and supporting volunteer programs. Indeed, given the problems with communicating across silos of services, it is better for any new supports to be connected to existing organizations and programs.

The literature reviews of other countries show that the difficulties identified by Aboriginal and CaLD people in this study are not unique to Western Sydney (5–8, 11). Migration to Western countries is expected to continue to increase due to conflict and climate change; and the First Nations people living in neo-colonial countries continue to struggle with disadvantage. However, the suggested strategies for amelioration are also applicable internationally. Countries with Western-developed medical systems all have the resources to meet the suggestions identified in this research. Implementing the flexibility of care evident in some palliative care units in other areas of the health service; developing trust; providing suitable care spaces; employing dedicated workers with requisite cultural and/or language skills; and developing community knowledge about EOL services are all realizable goals. However, they require the will to prioritize equity of access to quality EOL care.

### 4.3. Toward compassionate communities

The Compassionate Communities model advocates a move from current systems focused on mechanistic physiology and economics (21, 22) to local, civic, and corporate support initiatives for EOL. The aim here is to enable holistic supportive, palliative, and EOL care in and with the community where the patient and family are seen as people who are known and loved. The research results reflect the need for investing in a transition to Compassionate Communities (34, 52) via Rosenberg et al. (26) three principles: re-evaluation of organizational values; recognition of the primacy of caring networks; and realignment of the inherent paternalism in healthcare provision.

In re-orienting their organizational values, health services need to recognize that their assumptions are often not shared with those outside the health system. The adoption of Health Promoting Palliative Care would focus on relationships, respecting each patient as a whole person with their own lived experience and understanding of the world. Health providers, including non-specialists and, in some cases, specialist providers of palliative and EOL care are usually pressed for time and focus on their particular area of treatment. Often “Key Performance Indicator” checklists need to be followed so the service can demonstrate adequate care. Although such checklists can cover actions such as the provision of cultural sensitivity training, they would be challenged to cover the development of relationships and trust. Indeed, it would be unfair for an individual staff member to be held responsible for the lack of trust due to years of societal prejudice and mistreatment. However, there does need to be a palliative and EOL care service delivery model that promotes a relationship of trust, to support the quality care wanted by communities, and care that is not compromised by the suspicion that the person was being abandoned or “left to die” by a biased healthcare system. There needs to be EOL quality of care metrics that can evaluate the capacity of the service to support such relationships.

Most EOL caring takes place at home and social network maps for patients and caregivers depict medical services on the periphery or absent (53). It is, therefore, important for the model of care and the medical services to accommodate the reality that these are occasional contributors to a larger story about culture, community, friends, and family. The primacy of caring networks means that they cannot be set aside by patients and caregivers when the patient is in the hospital. Good connections rather than silos of care are needed so patients can move from home to the hospital when necessary and return without major disruption to their identity and support network. Assistance so cultures can maintain their rituals, provision of appropriate areas with art, music, kitchens, and space to gather, including outdoor space, can all help to support the patient's identity and therefore wellbeing.

By and large, individual healthcare staff are generally dedicated people doing good work often at some personal sacrifice. However, they operate in a system that promotes a paternalistic view of patients and their families. The system dynamic suggests that patients and families should not only be grateful for the care they receive but also accept that it is they who must adjust their expectations and behaviors when they have a problem with the medical system. These messages are not respectful. They do not engender trust. We need to reconsider, for example, whether the dominant narrative of pushing for individual decision-making and open communication and education about EOL is dismissing alternative values and beliefs. People holding different cultural perspectives are unlikely to easily adopt the values and worldview of the medical system.

Indeed, good caring is at odds with seeing people from the perspective of a mechanistic philosophy and public sector management framework that packages and standardizes healthcare in the name of “efficiency” at the cost of diverse individual needs (19). This approach typically guides EOL response and care in health systems across the Western world. However, the results from this research do show that people need guidance to interact in that “foreign territory,” preferably from trusted people who know both the patient's culture and the medical system. Such guidance needs to go beyond interpreting the words spoken. It cannot be assumed that people are familiar with palliative and EOL concepts and services, nor can it be assumed that they can, or think they should, ask for the help they need.

### 4.4. Limitations

The research was comprehensive in its ambitions to reach diverse voices and in its multi-method approach that laid a basis for the triangulation of the findings from different data sets. However, there were also limitations to the research design. The scope of the research was focused on a single local health district (LHD), albeit one with a large and particularly diverse population. This was relevant for this particular study, which was State government funded for the express purpose of conducting a culturally focused bereavement and palliative care needs analysis within the Western Sydney LHD. There is one dedicated palliative care unit within the LHD which is known for its progressive approach to culturally diverse supportive and palliative care. Expressed satisfaction with this service may limit the generalizability of some findings, but it also provides useful data about what works for people, and this was a key goal of the research questions. Furthermore, many participants had experiences with other palliative care units within Australia or their country of origin. Although comparative research across health districts would potentially add to the significance of the findings, the results are, nevertheless, of relevance both locally and internationally.

Participation in the research was limited to the Aboriginal community and the three largest CaLD groups residing within the LHD. This meant that some significant language groups were not allowed to contribute to the research. However, the target groups (Mandarin, Hindi, and Arabic) included in the research are the second, third, and seventh languages spoken worldwide (30), so what they have to say matters. A further limitation was that people were recruited for focus groups through LHD services. Again, while relevant for the project, hearing the voices of those using private or community “services” outside the public health system could potentially have contributed rich data about EOL experiences. The focus group participants were also limited to those who felt able to participate in face-to-face groups in the context of COVID. This no doubt prevented people, such as those with health vulnerabilities, from coming forward to share their views and experiences. Despite these limitations, the qualitative research generated important findings about the cultural needs of significant population groups, in breadth, if not in absolute participant numbers.

## 5. Conclusion

The changes in service culture and models of care indicated by this research are not necessarily low-cost or simple to implement. However, they are possible with a broader and more nuanced public debate on how diverse multicultural societies want to experience end-of-life and bereavement. The results from this study both prompt and contribute to such a debate. Many of the EOL challenges faced by culturally diverse people and groups can also be faced by those from the dominant culture who are not “death literate.” The range of issues raised here are all reasonable requests for bridging such a gap: knowledge of the systems and services; help discussing EOL needs in a respectful way; guidance in making decisions about medical treatment; performing EOL rituals that are important to us; having home comforts such as the food we like; and support of friends and family when in hospital.

Barriers due to language differences and past experiences of discrimination exacerbate the challenge of having one's needs met. Recognizing the needs of diverse and marginalized communities—such as the Aboriginal, Arabic, Mandarin, and Hindi-speaking groups in this research—is an important step toward understanding the diversity of needs and the challenges to managing EOL in a way that minimizes trauma and strengthens the bonds among people. It can be a time for friends and family to gather and connect and show respect to the dying person. The acceptance across society of a Compassionate Communities model would recognize the fundamentally social and cultural, rather than medical, nature of death, dying, and bereavement and remind us that they are, indeed, *everybody's business*.

## Data availability statement

The datasets presented in this article are not readily available because our ethics approval does not allow for this. Aboriginal people in particular are very cautious as they have been exploited in the past. Requests to access the datasets should be directed to r.leonard@westernsydney.edu.au.

## Ethics statement

The studies involving human participants were reviewed and approved by Western Sydney University Ref: H13743, Aboriginal Health and Medical Research Council of NSW Ref: 1657/20, and Western Sydney LHD Ref: 6530−2020/ETH00559. The patients/participants provided their written informed consent to participate in this study.

## Author contributions

RL had the major role in analyzing the data and writing the paper apart from the Method section which was led by the JP. JP also provided substantial input into the Introduction. PH integrated all the input from RL, JP, and SG and was responsible for checking references, formatting, and version control. SG provided significant input into the medical and palliative care system and services in the area. JT was responsible for project management, data collection, translations, and interpreters and transcriptions which were essential for this project. All authors contributed to the article and approved the submitted version.
